# Topics of Public Interest

**Published:** 1917-10

**Authors:** 


### TOPICS OF PUBLIC INTEREST

---

**Exemption of Medical Students.** It is announced that medical students who have attended colleges for one year or more will be subject to draft but that, if drafted, they will be allowed to continue their medical course—subject, probably to extreme military necessity—being considered as enlisted in the reserves. It is fair to say that this journal is one of very few in regarding the war as affording a favorable opportunity to reduce the number of physicians; and equally fair to state that our contention, based on statistic and other evidence, as to the existence of an over-supply of physicians, is not withdrawn. Military necessities will temporarily—for a period variously estimated at from a few months to several years—reduce the profession by about 20% and will also tend to diminish the graduation of students. The actual killing and disablement of military medical men will probably not be a marked factor; at the most, so far as can be judged from the experience of the other nations at war, it will amount to about 50% more than the normal depletion by death and incapacity in time of peace. Even if every medical officer were regarded as permanently removed from civil practice and if the exigencies of war removed every medical student, it would be at least five years before the problem of replenishing the medical profession would become acute. With all due respect, we beg leave to emphasize that the argument against drafting medical students and recent graduates, on the ground of need of internes, is not sound economically. We have become so used to the interne method of supplying public medical aid, that we have become blinded to the fact that it denotes an abnormal economic condition. Do not misunderstand us as implying that hospitals should not have a resident staff, or that the great majority of medical graduates should not have the benefit of this kind of practice, under responsible supervision, before engaging in private practice. The average medical graduate is 26 years old, he has had, at the very minimum, a high school education and from this up to a full college education; he has had four and possibly five years of intensive technical training. He is worth more than his board and laundry, with the possible addition of a hundred dollar check and a favorable acquaintance for a year's work, even if he does lack practical experience. He is worth just as much as a young engineer, clergyman, teacher, or even plumber or carpenter. If war conditions render it necessary for the taxpayers or the benevolent public to pay internes as any other young man, technically trained but inexperienced, is paid, it will be one of the best things that has happened for many years.

---

**Required Interne Service.** Six medical schools, all departments of universities, require a year of interne service before conferring the degree of M. D.: University of Minnesota,

Leland Stanford Jr., Rush (University of Chicago), University of California, Northwestern, University of Vermont. Pennsylvania and N. J. state laws already require a year of interne service and the following will require it: N. Dak. and R. I. in 1918, Ill. 1921, Mich. 1922.

---

**Administration of Anaesthetic by Nurse, Under Direction of Surgeon,** not a violation of law prohibiting practice of medicine by unlicensed persons. Ky. Court of Appeals, 194, S. W., 375. Frank vs. South.

---

**Expulsion From County Medical Society.** A voluntary association has the right to regulate the conduct of members and to expel but must do so in good faith and with provision for hearing. The expelled member must exhaust the remedies provided by the association before applying to a court of equity but may resort to suit after doing so or if the association does not provide for proper hearing. In the present case, the member was tried and convicted on the night on which charges were made; the trial was in violation of the by-laws of the society and the provisions for appeal were inadequate. Hence it was held that he had a right to apply to a court of equity for an injunction to restrain expulsion. Brown vs. Harris Co. Med. Soc., Texas Court of Civil Appeals, 194 S. W., 1179.

---

**Law Regarding Sexual Disease Advertising.** Section 1142 A of a law passed by the N. Y. legislature prohibits advertising concerning a venereal disease, lost manhood, impotency, seminal emissions, varicocele, self abuse, excessive indulgence, etc. The intent of the law is good but, as the Medical Record states editorially in its issue of July 28, it is so worded that it might be construed to apply to medical journals, as for example in the advertising of perfectly proper remedies. Moreover, while didactic or scientific treatises are exempted, as also advertisements and notices of incorporated hospitals, licensed dispensaries, municipal and the state department of health, there is a most unfortunate blunder—or possibly intentional qualification. The didactic or scientific treatises must not "advertise or call attention to any person or persons from whom or any office or place at which information, treatment, or advice may be obtained." Suppose that Dr. John Smith contributes a scientific article on any of the subjects mentioned, to a medical journal, adds to his name, as is customary, the statement of a professorship in venereal diseases or of a hospital or dispensary appointment, or of the authorship of a book, that, in accordance with a common sense rule, his city is appended and that, at the end of the article, likewise in accordance with precedent, his street address is given. This certainly calls attention both to the person and the place from whom at which information, treatment and advice may be obtained. Indeed, remembering the extensive and intensive ways in which laws in general are interpreted, it seems possible that specialization in genito-urinary and sexual practice may now be a criminal offense under the law of the state, at least unless the specialist takes particular pains to hide his light under a bushel.

In this connection, we may call attention to the federal liquor advertising law. As our readers have observed, we advocated that this law should, at the outset, have exempted medical journals and an analogous argument applies to the state law mentioned above. The principle involved is a broad one and susceptible of formulation: Any publication of a technical or scientific nature, maintained in good faith, should be allowed to publish any matter appropriate to its circulation. Matter, whether occurring in the reading or advertising pages that would be indecent for popular distribution, favors the cause of decency and morality when addressed to physicians, sociologists and others of similar training and intelligence. The discussion, even in an advertisement, of alcohol as a drug, inevitably calls attention to the fact that it ought not to be used as a beverage. Even the establishment of good faith is not especially difficult. It can easily be shown whether a medical or sociologic journal that discusses sexual problems, or alcohol, or narcotics or any other problem or thing that ought not to be brought too prominently to the attention of immature and untrained persons, is really distributed to the proper class or is catering to salacity or other improper tendencies and seeks a wide distribution.

---

**Reasonable Salaries for Public Medical Service.** Largely as the result of the efforts of the Chicago Medical Society (said to be the largest municipal medical society in the world), the Municipal Tuberculosis Sanitarium advertises the following schedule: All time service, 9 A. M.—5 P. M., first year salary $2,500, increasing $250 a year to $3,500 for the fifth and subsequent years.

---

**Open Hospital.** The new Chicago Lying-in Hospital, has been opened with a capacity of 120 beds. Any reputable physician may treat his patients in the hospital. We feel strongly that any hospital which is public in the receipts of funds, whether they come from taxes or from appeals to public benevolences, should be equally public so far as the recognition of the civic rights of physicians and of the mutual relation between physician and patient are concerned. And we hold it to be equally proper for any hospital to be private and to restrict the attendance on patients to certain physicians, so long as those interested secure the funds from their own pockets or from personal friends, or by the exercise of personal influence, so long as the institution is not represented as a public philanthropy. Our columns, as for any other question, are open to the expression of opinion.

---

**Railroad Accidents 1916.** Passengers killed 291, injured 8,008; Employees 2,941 and 176,923; Trespassers and others, 6,769 and 11,791. Total killed, 10,001, injured 196,722; excess over 1915, 1,371 and 34,835 respectively.

---

**Poliomyelitis.** At a conference with the Health Commissioner of Buffalo, Aug. 22, arrangements have been made for following up the cases of the 1912 and 1916 epidemics. Dr. Bernard Bartow will have general supervision of the work and cases will be operated upon at various hospitals or provided with prosthetic appliances, as required. It is estimated that there are about 100 children needing attention. During the epidemic of 1916, about 10,000 cases occurred in the state, more than 4,000 in July and nearly 6,000 in August. In 1917, 24 city and 17 upstate cases were reported in July and 14 and 30 respectively in August. No centers of infection are apparent either for the state or nation, this year. The press gives credit to the official representatives of the medical profession for the suppression of the epidemic. In one sense, we believe this credit is not deserved. The health authorities, with the support of the profession, took every possible precaution suggested by general principles, to suppress the epidemic of 1916 and with the result that the aggregate infantile mortality was lower than usual in spite of the epidemic but it may be questioned whether we know enough about the etiology of poliomyelitis to control it. As previously observed, sanitary and hygienic conditions seemed to have no bearing on the occurrence of cases and we might just as well admit that the relative cessation of the infection last year and its relative failure to reappear this year were either due to temporary exhaustion of vulnerable material or to some factor of which we have no knowledge.

---

**A Peculiar Effect of Food Conservation.** The City of Buffalo has been under contract to furnish the International Agricultural Corporation an annual minimum of 25,000 tons of garbage for reduction at $1 a ton. For the fiscal year ending June 30, only 19,000 tons were delivered and the corporation wants to be remunerated. Otherwise stated, a population of probably considerably less than 600,000 has consumed more than 6,000 tons of what in ordinary times would have been thrown into the swill tub; a utilization of about 20 pounds per capita per year, or of about 10 days' food supply.

---

**Zinc Oxid.** The Bureau of Chemistry, Dept. of Agriculture, reports that little C. P. ZnO is on the market. Most of the commercial samples were labeled "Not U. S. P.....Containing small quantities of lead."

---

**The U. S. Food Administration** announces the creation of two advisory committees as follows: Public Health, Leonard P. Ayer, Herman Biggs, David T. Edsall, Cary T. Grayson, A. Walter Hewlett, T. T. Janeway, F. G. Novy, Richard M. Pearce, H. Gideon Wells, Wm. H. Welch, Chairman. Alimentation, C. L. Alsberg, R. H. Chittenden, C. F. Langworthy, Graham Lusk, LaFayette B. Mendel, E. V. McCollum.

---

**Release of Medical Reserve Officers From Leases.** R. R. Denny of the Dennos Food Sales Co. is Chairman of a committee of the Chicago Rotary Club to investigate cases in which physicians who have volunteered for military duty are being held by landlords to the terms of leases. While comparatively few cases of lack of patriotism have been encountered, about 100 complaints are being investigated. Anyone interested is invited to communicate with Mr. Denny.

---

**Civil Service Vacancies.** We have received announcements of more or less desirable openings, too late for publication. As this is a frequent occurrence, we would advise those of our readers who are desirous of obtaining such positions, to notify us in advance, so that we may notify them by telephone or letter of future opportunities.

---

**Suicide Statistics.** The Metropolitan Life Insurance Co. states that among four million white male industrial policy holders, 620 suicides occurred in 1916, the rate being 15.3 per 100,000 as against 19.6 for 1915. Among nearly five million females, the rate was 6.3 for 1916, as against 7.5 for 1916. Nearly a third of male suicides were by fire-arms and more than a third of female suicides by poison. Among negroes, the rates for 1916 were 8.2 for males and 3.4 for females, also for industrial workers. Male policy holders show a lower suicide rate than the population generally up to the age of 25, above that the rate is slightly higher. Female policy holders show a lower rate than the general population throughout.

---

**U. S. Civil Service Positions.** The U. S. Civil Service Commission, Washington, announce that several positions are open in the Indian Service at $1,000-$1,200, Panama Canal Service at $1,800, Public Health Service $480 for part time—$1,800 for whole time, Coast and Geodetic Survey $1,020 and subsistence allowance while on board ship at $1-$2.50 a day. No formal examination will be held but physical ability will be rated as 10 points, education, training and experience as 90 points. The requirements vary somewhat and for certain positions, senior medical students may qualify in advance of graduation.

---

**Sale of Niagara Medical College Building.** The old building on Ellicott Street, Buffalo, has just been sold. It is interesting to note that at the meeting of the stockholders, only one physician was directly involved, and he was not a member of the original faculty, all the rest being represented by their heirs, in some cases, two degrees removed. The first class graduated was 1886, the last, 1898. After this date, the school was merged with the Medical Dept., University of Buffalo.

---

**Birth Registration Area.** This was established in 1915, comprising the District of Columbia and 10 states, 10% of the area and 31% of the population of the country. Md., Va. and Ky. have recently been added and the attention being directed to the matter by military conditions is expected to bring 2/3 of the population within the area within the next two years. The fact that birth statistics can be collected retro-actively is indicated by the fact that a single physician, stimulated by the importance of accurate records in connection with the draft, has reported 450 births which have occurred in his practice since 1900.

---

**Mayo Foundation.** The regents of the University of Minnesota ratified unanimously, Sept. 15, the provisional agreement by which the Mayo Foundation at Rochester becomes the absolute property of the University, in perpetuity for higher medical education, research and investigation. Securities totaling $1,650,345 were turned over to the University by Mrs. Wm. J. and Chas. H. Mayo.

**Buffalo School Statistics.** The registration figures of this year as compared with last year are:

| High Schools. | 1917. | 1916. |
|---|---|---|
| Hutchinson | 1,498 | 1,593 |
| Lafayette | 900 | 982 |
| Masten Park | 918 | 955 |
| Technical | 632 | 828 |
| South Park | 702 | 698 |
| Totals | 4,650 | 5,056 |
| Decrease, 406. | | |
| Vocational Schools. | | |
| Elm | 116 | 92 |
| Seneca | 103 | *... |
| Peckham | 215 | *... |
| Black Rock | 60 | *... |
| Total | 494 | |
| *No report of attendance for 1916. | | |
| Grammar Schools | 46,087 | 45,916 |
| School of practice | 292 | 290 |
| Teachers' training school | 58 | 58 |
| Truant school | 3 | 0 |

The 62 elementary Roman Catholic schools last year had a registration of 26,031 while 1,629 were enrolled in private schools. The population of school age for the school year 1916-17 was 105,096, distributed according to language of parent as follows: English 46,334; German 21,331; Polish 22,878; Jewish 3,238; Italian 8,616; Scandinavian 653; Hungarian 1,120. 20 other nationalities 926.

---

**Soy Bean—A Dangerous Statement.** One of our contemporaries prints in the tabloid form of statement that is so apt to be copied widely, the advice that as this bean contains no starch, it is a valuable food for diabetics. How about the 9% of saccharose?

---

**Food Supply of the U. S. Sept. 1.** The Bureau of Markets gives the following statistics, which represent the reports of large concerns (number for each food shown in parenthesis) but not the absolute totals. Frozen beef (236) 116 million pounds; Cured beef (293) 31 million pounds; Frozen lamb and mutton (149) 3 million pounds; Frozen pork (259) 75 million pounds; Dry salt pork (389) 197 million pounds; Sweet pickled pork (481) 318 million pounds. (We suppose that this refers to pork temporarily preserved in brine so that it can be used as fresh pork. There certainly can be no such enormous quantity of pork made into what is ordinarily called sweet pickle). Lard (482) 97 million pounds; Frozen poultry (482) 97 million pounds; Creamery butter (335) 99 million pounds; Packing stock butter (82) 2½ million pounds; American cheese (373) 76 million pounds; Eggs (400) 6 million cases; Frozen eggs (148) 17 million pounds. It will be noted that these foods represent animal proteid and fat and, at a rough estimate, with due allowance for the substitution of one kind of proteid or fat for another according to supply available, the figures quoted represent about a month's supply for the whole country. This statement does not mean that every one can take all the fat he wants in the form of butter nor that the supply represents a month's rations in calories, without reference to the normal major use of vegetable proteid from cereals, and the calories derived from starch in the same cereals or other vegetable foods, sugar, etc. What is still more significant and encouraging, is that, so far as can be judged from the statements of firms reporting for the present and last year, there has been a considerable increase in most of the foods on hand.

---

**The American Ambulance** at Neuilly, near Paris, was openen in Lyceé Pasteur, Aug. 14, 1914 with 160 beds and a number of improvised ambulances made from Ford Chassis and packing boxes. It grew to 600 beds, nearly 100 ambulances and had 170 men in the ambulance corps. It was officially terminated July 22 with appropriate ceremonies, the equipment being turned over to the American Red Cross as American Army Hospital No. 1, under charge of Major Geo. P. Peed, M. D., U. S. Army.

---

**Medical History of War.** Col. C. C. McCullock, Librarian of the Army Medical Library, Major F. H. Garrison, Asst. Librarian and Capt. John S. Fulton, Sec. of the Maryland State Board of Health, have been appointed by Surgeon-General Gorgas, to prepare the medical history of the present war. Major Garrison will write the text and Capt. Fulton will collect statistics. This prompt action is in line with that already taken by other countries. The History of the Civil War, in 6 large volumes, was not completed till 28 years after the war so that it was almost valueless except as history. It is the intention to issue the history of the present war with as little delay as possible, so as to render it available for practical medical and surgical purposes.

---

**Reconstruction Hospitals.** While in former wars, military medical responsibility ended with the discharge of the wounded soldier after his wounds had healed in the immediate sense, the present idea is to extend the responsibility to the restoration of health and function to the maximum degree possible. Institutions for this purpose will be established at Boston, N. Y., Washington, Chicago (these four expected to be the first completed), Philadelphia, Baltimore, Buffalo, Cincinnati, St. Paul, Seattle, San Francisco, Los Angeles, Denver, Kansas City, St. Louis, Memphis, Richmond, Atlanta, New Orleans. These will be, to a large degree, orthopaedic hospitals but will also include provisions for manual training, re-education, use of prosthetic appliances, training in the use of special instruments and machines designed to supplement physical handicaps. The ultimate purpose is to return the maimed soldier to civil life so that he can engage in his former occupation or some substitute, without the economic handicap which has formerly affected the victims of war. Arrangements will be made with outside industries for training with special machinery when this can be done better than at the institutions themselves and an employment bureau will be established. At the outset, each hospital will accommodate 500, the number to be increased to 1000 as necessary. This whole matter comes under the department of military orthopedic surgery recently organized in the Medical Department of the Army. The following officers of the Medical Reserve Corps are in charge of the work: Maj. Elliott G. Brackett, of Boston, director of the department of military orthopedics to the Surgeon General; Maj. Joel E. Goldthwait, of Boston, director of military orthopedics for the expeditionary forces; Maj. David Silver, of Pittsburgh, assistant director of military orthopedics to the Surgeon General. The following, in conjunction with the above staff, compose the orthopedic council: Dr. Fred H. Albee, of New York; Dr. G. Gwilym Davis, of Philadelphia; Dr. Albert H. Freiberg, of Cincinnati; Dr. Robert W. Lovett, of Boston, and Dr. John L. Porter, of Chicago.

The Dept. of Military Orthopaedic Surgery will begin its work with patients near the firing line and will have special base hospitals in Europe. It will also provide for instruction of assistants in the encampments in this country and those specially fitted will take part in the examination of recruits and their rehabilitation when possible. Semi-monthly inspections of the feet will be included in this special work. A manual or military orthopaedic surgery will soon be ready for distribution.

---

**Claims of Approval** of food stuffs or their prices, by the Food Administrator, have been made or implied by certain firms, in their advertising. Mr. Herbert C. Hoover denies that any such endorsements have been made, and will serve injunctions to stop the practice, if necessary. This is a reminder of the attempt to use the serial numbering of the Food & Drug Law as a guarantee by the government.

---

**Information in re Medical Reserve Corps.** The recommendations and acceptances for the different branches of the Medical Department to Sept. 17, 1917, are as follows:

| | Recommendations. | Acceptances. |
|---|---|---|
| Medical Reserve Corps | 15,600 | 11,156 |
| Dental Reserve Corps | 4,004 | 2,491 |
| Veterinary Reserve Corps | 1,250 | 725 |
| Sanitary Corps | 277 | 146 |
| Ambulance Corps | 24 | 5 |
| Total | 21,155 | 14,523 |

In regard to the Reserve Corps and the Red Cross. Medical men may attach themselves to the Red Cross and be assigned to strictly Red Cross duty. If so, they occupy the status of civilians and have no connection whatever with the Army. On the contrary, those who are assigned to Red Cross Base Hospital Units for instance, must be commissioned in the Medical Reserve Corps, and occupy exactly the same relation to the Department as any other officer of the Medical Reserve Corps. They are subject to such assignment as the Department finds necessary, although naturally they will be retained on duty with their unit unless unforeseen circumstances arise which make their separation from it necessary. The officers who are on strictly civilian duty with the Red Cross are, of course, not paid by the Government and do not wear military uniforms.

R. B. Miller, Lieut. Col., Medical Corps, U. S. Army.

By direction of the Surgeon General.

Note: The figures kindly supplied by the Surgeon General's Office, have an important bearing on the proposed extension of the draft of medical men as such. It will be noted that about 75% of the total number of medical reserve officers estimated as necessary have actually been recommended for commissions. Considering that there is inevitably some delay between the actual time of volunteering and the completion of examination, passing upon reports, etc., preliminary to recommendation to the Adjutant General for commission, (in a case personally known, amounting to two months) and that not all volunteers are eligible, it may be assumed that as many men have volunteered as would have been called by draft. Reference to the second paragraph implies also that a considerable additional force has been raised for Red Cross work, aside from the Medical Reserve Corps proper. The criticism has been made that, after volunteering, physicians do not accept commissions. While this breach of courtesy, to say the least, has occurred, it should be remembered that about two weeks must elapse between the recommendation for and the receipt of acceptance of a commission and it has been stated that 100-150 applications are being received daily so that the acceptances at any rate are somewhere about 1500-2000 behind the recommendations. The fact is that the volunteer system for medical officers has already supplied the demands of the regular army and national guard (some 800,000 troops), provided or anticipated much of the estimated requirement of base hospital service, with a reserve nearly or quite adequate for the first draft. We do not oppose the drafting of medical men as a future expedient but believe that the decision of this question should rest solely with the proper authorities. We feel, however, that, with due allowance for inevitable delays, personal and official, the medical profession has made good on the volunteer system.

---

**The Meanest Man.** We enter in the contest for the prize, those who have taken advantage of the patriotism and industry of girls, to raise the price of knitting needles to an exorbitant standard.

